# Effects of Multipath Attenuation in the Optical Communication-Based Internet of Underwater Things

**DOI:** 10.3390/s20216201

**Published:** 2020-10-30

**Authors:** Rabia Qadar, Waleed Bin Qaim, Jari Nurmi, Bo Tan

**Affiliations:** 1 Unit of Electrical Engineering, Tampere University, 33720 Tampere, Finland; waleed.binqaim@tuni.fi (W.B.Q.); jari.nurmi@tuni.fi (J.N.); bo.tan@tuni.fi (B.T.); 2 DIIES Department, Mediterranea University of Reggio Calabria/CNIT, 89122 Reggio Calabria, Italy

**Keywords:** scattering, multipath attenuation, magnitude response, temporal dispersion, bit error rate, Internet of Underwater Things (IoUTs)

## Abstract

The propagation of light underwater is tied closely to the optical properties of water. In particular, the underwater channel imposes attenuation on the optical signal in the form of scattering, absorption, and turbulence. These attenuation factors can lead to severe spatial and temporal dispersion, which restricts communication to a limited range and bandwidth. In this paper, we propose a statistical model to estimate the probability density function of the temporal dispersion in underwater wireless optical communication (UWOC) based Internet of Underwater Things (IoUTs) using discrete histograms. The underwater optical channel is modeled using Monte Carlo simulations, and the effects of temporal dispersion are presented by measuring the magnitude response of the channel in terms of received power. The temporal response analysis is followed by an extensive performance evaluation in terms of bit error rate (BER). To facilitate in-depth theoretical analysis, we have measured and presented magnitude response and BER of the channel under different field-of-views (FoVs), apertures, and water types. The three main areas under study are (i) BER versus link distance behavior, (ii) temporal response of the channel, and (iii) effect of scattering on photon travel. Our study shows the two main factors that contribute to beam spreading and temporal dispersion are (i) diffusivity of the optical source and (ii) multiple scattering. Furthermore, our results suggest that temporal dispersion caused due to multiple scattering cannot be mitigated completely; however, it can be minimized by optimizing the receiver aperture.

## 1. Introduction

**Figure 1:**
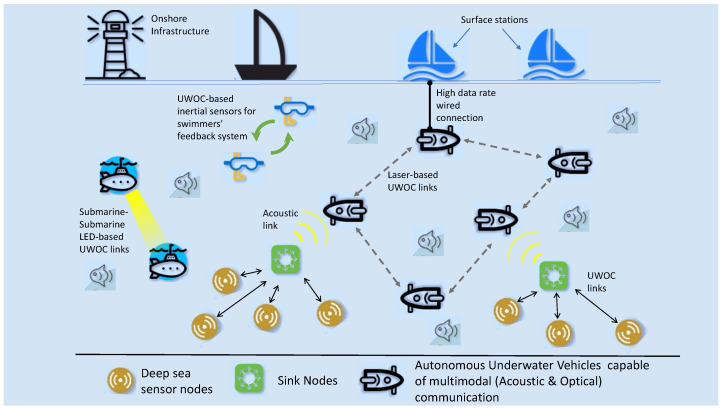
A general Internet of Underwater Things (IoUTs) framework.

Internet of Underwater Things (IoUTs) is an effective means of replicating human capability of collecting and processing data from vast, hazardous, and deep underwater environments. It has been made possible by deploying sensor nodes that perform the task of distant real-time sensing, collecting, processing, and transmission underwater. Although the field of sensor technology has developed tremendously in the last few years, the participating sensor nodes are still likely to fail because of limited resources, dynamic network topology, scalability, various traffic types, and unreliable underwater environment. There has been an increasing trend in deploying IoUTs to enable different underwater applications (depicted in Figure 1) such as monitoring, surveillance, offshore operations, military activities, sports, entertainment, and tourism [1]. One of the sophisticated research efforts and diverse solutions for the implementation of these activities is underwater wearables [2,3,4,5,6]. Along with the ability to sense, operate, and communicate, underwater wearables require novel networking concepts that are key to reliable means of exchanging information. The optical communication based underwater wearables target short-range oceanic applications, where the depth and transmission distance range up to a few hundred meters [7,8]. However, these networks must also consider harsh environments with limited bandwidth, high scattering, and absorption effects that cause temporal dispersion and inter-symbol interference (ISI) [9].

Maintaining optical communication underwater is a crucial task, and it is not feasible to design a communication link with accurate pointing and required performance. That is because of the numerous constraints induced as a result of channel effects. Absorption and scattering are two diverse effects of the same phenomenon, i.e., interaction of light photons with particulate matter and water molecules, leading to path loss, deviation of photons, and degradation in received power [10]. These inherent optical properties of water (i.e., absorption and scattering) can severely contribute to time-varying multipath effects. These multipath effects can be categorized into spatial and temporal dispersion. Spatial dispersion is caused by the spreading of light beam as a result of multiple scattering, consequently reducing the number of photons at the receiver. If light is diffused by nature, spatial spreading may also occur due to initial transmitter beam distribution apart from channel effects [10,11]. Temporal dispersion, on the other hand, causes the photons to take longer to reach the receiver, thus increasing time delays and path length differences.

Effects such as absorption can be controlled by (i) operating in the black-green portion of the visible light region, (ii) by using efficient photon receivers, or (iii) by increasing the transmit power [12]. However, effects such as scattering that cause spatial and temporal dispersion can essentially limit the optical communication range and bandwidth. Since scattering is not directly controlled, its effects can be minimized by optimizing the essential system parameters such as FoV, link separation, and aperture. Therefore, it is of key importance to characterize the underwater optical channel and set the essential system parameters to enable high-quality communication. In this paper, we carry out a detailed study on the sources of temporal dispersion in harbor, coastal, and ocean water for underwater wearables operating in shallow waters where optical wireless communication is limited to a few centimeters. Our main contributions can be summarized as follows:

- We designed a unified system model that accommodates channel loss due to multiple scattering and characterized its respective performances in terms of magnitude response for different system configurations (FoVs, apertures) and water types (clear ocean, coastal, and turbid harbor).
- We proposed a Monte Carlo-based statistical model that utilizes a discrete histogram of the total received power to characterize temporal dispersion.
- Based on our model, the respective BER and magnitude responses resulting from the temporal dispersion under different FoVs, apertures, and water types are simulated and presented.

The rest of the paper is organized as follows. In Section 2, we discuss the existing methods of temporal dispersion modeling underwater. In Section 3, we provide analytical details about the simulated underwater environment and our proposed method. Section 4 explains our findings on various system configurations and parameters, and Section 5 compares our methodology. Section 6 concludes our work and suggests future research directions.

## 2. Related Work

Several studies have demonstrated the characteristics of the underwater optical channel through experimental, analytical, and statistical methods. Because of the difficulties in performing experiments in an incredibly dynamic and rigorous undersea environment, most of the experimental work is carried out inside a controlled laboratory setup. An experimental approach for measuring the frequency response of the underwater laser communication channel under the influence of scattering has been implemented in [12]. They concluded that temporal dispersion is insensitive to increasing FoV over loose pointing and tracking, but increasing FoV can improve the signal-to-noise ratio (SNR) and dynamic range. The authors in [13] performed an experimental study of the underwater optical channel by measuring the magnitude and phase response of an underwater optical channel for 25 attenuation lengths in harbor waters. Most recently, the authors of [14] validated their experimental investigations of the light propagation underwater through Zemax ray-tracing software. However, the authors of [15] validated a Monte Carlo-based model against an experimental model that can predict temporal and spatial dispersion in underwater optical laser-based links.

To overcome the constraints of the laboratory-based experimental study, researchers have implemented analytical methods of estimating the underwater optical communication link. While investigating the effects of spatial spreading on the optical beam, a hypothetical model for the characterization of beam spreading of the underwater wireless optical communication (UWOC) channel was proposed in [16]. They validated their theoretical predictions through a series of lab experiments. Additionally, the total scattering profile, i.e., temporal and spatial spreading of a light beam, was investigated in [17] as a function of water turbidity and link range. The proposed theoretical model is simple and requires less computation time because of the use of Small-Angle Approximation (SAA). Considering the effect of light polarization along with multiple scattering, an analytical solution was proposed in [18] using the Stokes Vector.

Analytical methods are not an ideal approach for evaluating underwater communications for practical operations since they involve a chain of assumptions and simplifications. While considering the random nature of the underwater wireless communication, several researchers have used the well-known Monte Carlo simulations to study the response of the underwater channel for different water types, system configurations, and propagation distances. The authors in [19] characterize the spatial and temporal behavior of the underwater channel by measuring IR and path loss considering single, multiple, and no scattering using a stochastic analytic approach. A numerical model of the impact of scattering on light beam underwater was proposed in [9] using the Monte Carlo technique. They analyzed the IR and received signal power of the UWOC channel against receiver FoVs and apertures. Using the Monte Carlo approach, the research in [20] models an underwater optical channel. They quantify the channel’s IR to deduce information of attenuation and temporal dispersion for different lens diameters, link distance, and water types. Authors in [21] evaluated the influence of temporal dispersion on UWOC performance through channel’s IR and BER analysis. They implemented maximum likelihood sequence estimation (MLSE) to mitigate ISI induced due to limited bandwidth. For a varying FoV, the authors of [11] evaluated the distribution of power and frequency response for a short-range on-axis and off-axis line-of-sight (LoS) link using a Monte Carlo model. They suggest that smaller FoV can facilitate to improve power performance for the ocean and coastal water. However, for harbor water, a large FoV is required to maximize received power with sufficient bandwidth. Table 1 summarizes the major advantages and limitations of the analytical, experimental, and simulation-based methods of characterizing UWOC.

### Motivation

As discussed previously, several studies have addressed the channel characterization and temporal response modeling of UWOC links. The Monte Carlo-based simulation method can provide the required precise representation of the underwater channel with high resolution as compared to experimental and analytical methods given that it uses the exact volume scattering phase functions and absorption coefficients [22]. It is also the most widely used technique for predicting the causes and effects of temporal and spatial dispersion in UWOC. In this work, we have designed a simple, scalable, and comprehensive statistical model based on Monte Carlo simulations. Our proposed model forms impulse responses of the laser-based underwater wireless optical communication link by first characterizing the channel and then generating a discrete histogram of the total received power. This histogram contains the received photons which are scattered more than once and have experienced absorption of varying degrees. We explore the temporal spreading of light with more realistic underwater conditions. Therefore, we use the Petzold’s measured volume scattering phase functions to simulate multiple scattering. Additionally, we perform simulations to determine the performance of such systems in three different water types in terms of BER and average distance traveled by photons.

## 3. Propagation Model

We consider a statistical approach for modeling the underwater channel. The goal of this study is to provide a comprehensive and scalable model that provides channel estimation and performance study for the three major types of water, i.e., harbor, coastal, and ocean. The model estimates the channel by considering light as a collection of photons and tracks every photon propagation from the transmitter to receiver using the Monte Carlo method.

### 3.1. Model Requirements

Our technique illustrates the photon transmission as a probability distribution that describes the path length of the photon propagation before a photon interacts with a particle and the angles of scattering after a scattering event occurs. Since our model incorporates the Monte Carlo approach, which is statistical in nature, it relies on calculating the radial angles and directions of a large number of photons (usually $10^{5}$). Our method tracks photon propagation through water by repeatedly and randomly choosing a random number originator to generate bits in the interval (0,1) and random angles between (0 and 2π). However, in underwater communications, due to limited knowledge about the transmission, reception, and channel conditions, we have made certain assumptions as follows:

- no shift in frequencies of the incident and scattered light is considered, the only change that is considered is the change in direction;
- the phase functions are used to measure the anisotropy of scattering;
- scattering and absorption are uniform throughout the channel;
- the boundaries are absolutely absorbing, and any photon traveling past the receiver or transmitter plane is marked as terminated.

Our simulation model comprises the following three stages

1. the photon’s initial conditions,
2. light transport under water, and
3. photon reception.

#### 3.1.1. Photon Initial Conditions

The initial conditions for the light source are simply considered of an ideal light, which is collimated in nature, e.g., a laser diode. All photons are initialized with same locations, directions, and unity weight. However, for a real source, the conditions will vary to some degree and should be included in the light beam profile. The starting x,y location of the photon is simulated using initial radius $r_{0}$ and radial angle ϕ, such that

(1) $$\begin{array}{cc} x_{0} & =r_{0}\cos\phi \end{array}$$

(2) $$\begin{array}{cc} y_{0} & =r_{0}\sin\phi \end{array}$$

and starting direction using initial divergence and radial angles,

(3) $$\begin{array}{cc} \mu_{x} & =\sin\theta_{0}\cos\phi \end{array}$$

(4) $$\begin{array}{cc} \mu_{y} & =\sin\theta_{0}\sin\phi \end{array}$$

(5) $$\begin{array}{cc} \mu_{z} & =\cos\theta_{0} \end{array}$$

#### 3.1.2. Light Transport Underwater

The a priori known variables given to the model are the underwater coefficients, i.e., water albedo $w_{o}$, attenuation coefficient *c*, and volume scattering phase function γ. The albedo is the ratio of scattering coefficient *b* in m${}^{-1}$ to the attenuation coefficient *c*, also known as the extinction coefficient. It defines the total loss of energy as a sum of absorption and scattering coefficient c(λ)=a(λ)+b(λ). Moreover, the attenuation coefficient is also wavelength dependent. Different attenuation coefficient values determine the amount of attenuation the optical signal will face underwater. Table 2 lists Petzold’s measured water albedo and attenuation coefficients for the three water types. The simulation model is based on a Cartesian coordinate system with photons traveling along the *z*-axis. The initial location of the photon is determined by the x,y,z cartesian coordinates; whereas, the direction of the photon is described by the direction cosines $\mu_{x},\mu_{y},\mu_{z}$. Instead of calculating the probability of absorption at every event (i.e., scattering or boundary interaction), we suppose that absorption would have occurred; hence, the weight of the photon is updated using the relation

(6) $$\begin{array}{cc} w_{n+1} & =w_{n}*\left(1-\frac{a}{c}\right) \end{array}$$

where *a* is absorption coefficient in m${}^{-1}$ and *c* is attenuation coefficient. To simulate scattering, we utilize scattering phase functions measured by Petzold [23], given as

(7) $$\begin{array}{cc} \gamma (\lambda ,\psi ) & =\frac{\phi_{s}\left(\psi ,\lambda \right)}{\phi_{i}\left(\lambda \right)l\Delta \Omega }. \end{array}$$

where $\phi_{s}\left(\psi ,\lambda \right)$ is the power scattered into the solid angle Ω after leaving the sample volume of length *l* at an angle ψ with respect to the incident power $\phi_{i}\left(\lambda \right)$. The total volume scattering function is given by

(8) $$\begin{array}{cc} b & =2\pi \int_{0}^{\pi }\gamma \left(\lambda ,\psi \right)\sin\theta d\theta . \end{array}$$

**Figure 2:**
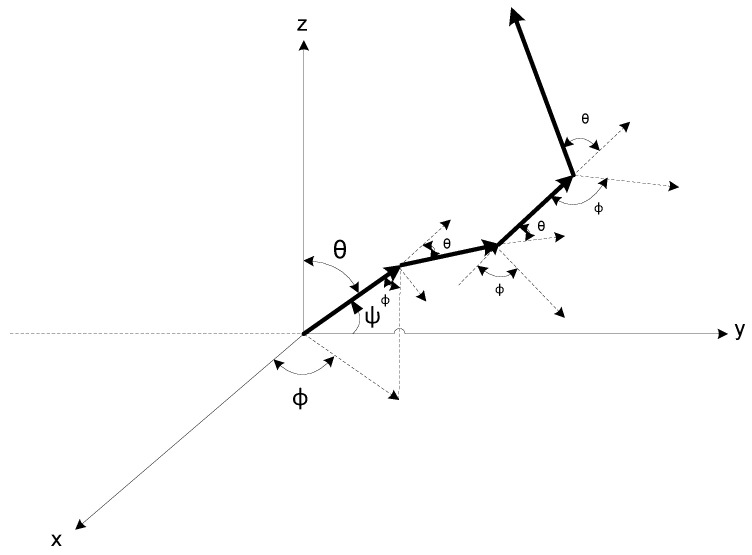
Illustration of photon travel, where θ is the scattering angle determined using [23], and ϕ is the azimuth angle [0, 2π].

We implemented the anisotropic scattering (see Figure 2) model in which scattering angles are determined using Petzold’s phase functions [23]. They help to study temporal spreading in detail and give a more realistic channel response, as compared to a Beer–Lambert model which is a rather simple but unrealistic approach. Thus, when we closely study scattering, we come across different characteristics of water that indicate significant aspects of channel losses, as discussed in Section 4.

Photon position and direction update whenever it hits the water surface (or any particle) and when it reaches the receiver and has not yet been marked as received or terminated. The successive position of the photon is determined using the pseudo-random path length *‘l’*. The path length is the distance a photon travels without being scattered or absorbed and is calculated at the beginning of the simulation. The variables $x^{\prime },y^{\prime },z^{\prime }$ are the updated positions, $x_{0},y_{0},z_{0}$ are the old positions (before scattering event) and $\mu_{x},\mu_{y},\mu_{z}$ are initial directions.

(9) $$\begin{array}{cc} x^{\prime } & =x_{0}+\mu_{x}l, \end{array}$$

(10) $$\begin{array}{cc} y^{\prime } & =y_{0}+\mu_{y}l, \end{array}$$

(11) $$\begin{array}{cc} z^{\prime } & =z_{0}+\mu_{z}l. \end{array}$$

Given the scattering angles θ and ϕ and the initial directions $\mu_{x},\mu_{y},\mu_{z}$, we also update the directions. If the photon is close to the *z*-axis, i.e., $|\mu_{z}|>0.999$, then the direction cosines are updated according to [24]

(12) $$\begin{array}{cc} \;\mu_{x}^{\prime } & =\sin\theta \cos\phi , \end{array}$$

(13) $$\begin{array}{cc} \mu_{y}^{\prime } & =\sin\theta \cos\phi , \end{array}$$

(14) $$\begin{array}{cc} \mu_{z}^{\prime } & =sign(\mu_{z})\cos\theta . \end{array}$$

#### 3.1.3. Photon Reception

The photon propagation cycle, i.e., path length → photon scattered → weight drops → coordinates update, repeats until it reaches the following instances:

1. Photon’s remaining weight/power is negligibly small, in which case the weight is rouletted [25].
  (15) $$\begin{array}{c} w_{n+1}=\left\{\begin{array}{cc} w_{n}\tau & \;\;\mathrm{if}\;R\leq \frac{1}{\tau }\\ 0 & \;\;\mathrm{if}\;R>\frac{1}{\tau } \end{array}\right.. \end{array}$$
  where $w_{n+1}$ is the weight after scattering, and $w_{n}$ is the old weight. *R* is the uniform random variable in the interval [0, 1], and τ is the roulette threshold. If *R* is less than the fraction 1/τ, then the old weight is scaled roulette threshold times, and if it is greater than the fraction, the weight tends to zero which means the photon is completely absorbed. This method limits the number of computations and also preserves the total probability.
2. If the photon hits a boundary other than the air–water interface (in such case the photon continues to propagate), it is terminated.

We recommend the readers to refer to our previous work [10] for further details of the propagation model.

### 3.2. Temporal Response of the Channel

We design a unified system model that accommodates channel loss due to multiple scattering and measures its respective performances in terms of magnitude response for different system configurations and water types. Depending upon the degree of spatial dispersion and system variables, the scattered photons incur distinct path lengths. These path length differences between photons give rise to temporal dispersion. In addition to the spreading of light, it also causes loss of received power, resulting in reduced signal levels.

We first estimate the channel using a combination of water parameters including water albedo $w_{o}$, absorption, scattering coefficients, and system parameters, i.e., receiver FoV, aperture, and link distance. Our simulations track each photon path for a homogeneous medium to generate photon time-of-flight (ToF). ToF is a measure of the speed of photon propagation under the water. It is directly proportional to the product of photon distance traveled $d_{pro}$ and refractive index of water $n_{w}$; however, it is inversely proportional to the speed of light in vacuum *c*, as given in Equation (16):

(16) $$\begin{array}{c} t_{ToF}=\frac{d_{pro}n_{w}}{c}. \end{array}$$

**Figure 3:**
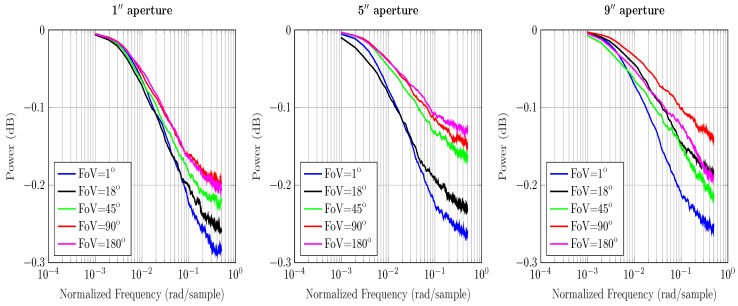
Comparison of the magnitude response of ocean water under various aperture sizes and FoVs for a 3 m LoS link.

**Figure 4:**
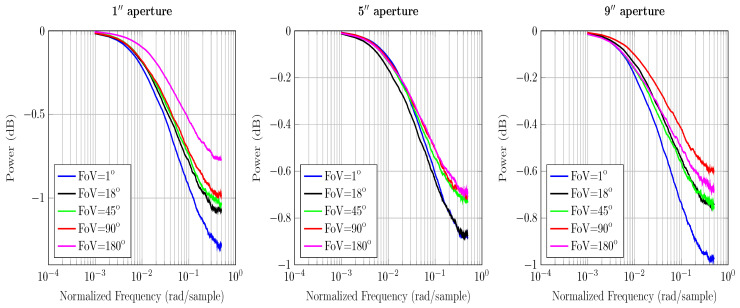
Comparison of the magnitude response of coastal water under various aperture sizes and FoVs for a 3 m LoS link.

**Figure 5:**
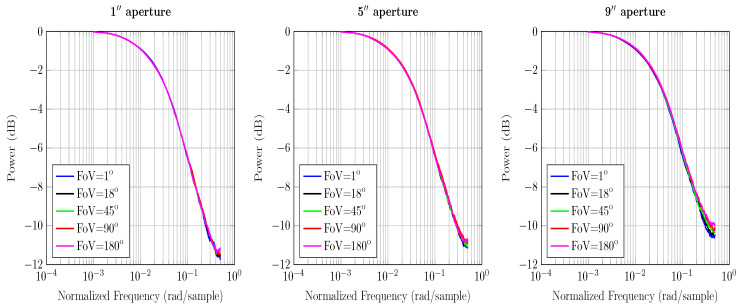
Comparison of the magnitude response of harbor water under various aperture sizes and FoVs for a 3 m LoS link.

The ToF equation gives information about channel’s temporal response and its frequency. In order to determine the discrete time channel IR in terms of the received power, we first form a histogram of the total received power and then normalize it by the weight of the histogram, i.e., the total number of received photons. In principle, impulse response gives the dispersal of power over time at the receiver. For an equivalent magnitude response, we simply take the z-transform of the IR and arrive at Figure 3, Figure 4 and Figure 5.

#### Discrete Histograms and Their Respective Bin Widths

The histogram is a conventional method of estimating the densities of ordinal data sets. It also offers a true estimate of the probability density function. For the least mean squared error, the appropriate choice of bin size is of utmost importance. If the bin size is too small, the histogram will be too uneven; conversely, if the bin size is too large, the histogram will be too flat, equivalent to a large variance. The appropriate choice of bin size not only balances the variance, but it also minimizes the mean squared error. For a fairly large data set such as the received number of photons, it is crucial to obtain the optimal number of bins to place all the photons’ received powers in the appropriate bins based on the time-of-flight information. Therefore, for the ideal number of bins and respective bin sizes $t_{bw}$ we use Scott’s rule [26]. The optimal bin size requires an estimate of the true elemental density *f*; however, Scott [26] derived an equation for the optimal choice of histogram bin size that either uses the knowledge of the density function *f* or determines the bin width based on normally distributed data.

(17) $$\begin{array}{cc} h_{n} & ={\left\{\frac{6}{f^{\prime }{\left(x\right)}^{2}dx}\right\}}^{\frac{1}{3}}n^{-\frac{1}{3}}, \end{array}$$

(18) $$\begin{array}{cc} h_{n} & =3.49\sigma n^{-\frac{1}{3}}, \end{array}$$

where σ is the estimated standard deviation, and *n* denotes the sample size. Although the normal $\left(Gaussian\right)$ density of data forms the basis for Equation (18), actually it is not as strong as the constant normal assumption, i.e., use of Equation (18) on non-normally distributed data, will not result in a histogram that looks like normal. The bin size $t_{bw}$ also determines the maximum frequency of the channel due to the reciprocal frequency–time relationship. Therefore, we can say the maximum frequency that can be represented in estimating the channel time dispersion is derived as

(19) $$\begin{array}{c} f_{max}=\frac{c}{t_{bw}n_{w}}. \end{array}$$

where *c* is the speed of light in vacuum, $t_{bw}$ is bin size, and $n_{w}$ is the refractive index of water.

## 4. Simulation Results

In this section, we present the simulation results for the temporal response and BER performance of a LoS UWOC system in the presence of multipath attenuation. Table 3 lists the coefficients, symbols, and associated values for all the important parameters used for temporal dispersion characterization and MC-based channel simulation. The system consists of a single transmitter and a receiver established in three different water types, namely harbor, coastal, and ocean. In our simulations, we have considered a 3 m device-to-device distance, keeping in view the highly scattered nature of underwater channels and LoS conditions. The optical beam is modulated and detected symbol-by-symbol using Intensity Modulation/Direct Detection (IM/DD). All photons have been transmitted initially using the same transmit power, i.e., unity, and the same beam divergence of 1.5 mrad. On the receiver end, the apertures and FoVs are varied for depicting various device sizes. Although, accurate pointing and tracking is not fully achievable underwater, but with the use of a collimated light beam, the LoS geometry is not affected by severe temporal dispersion to the point where multiple scattering dominates. This is why we see a lower degree of temporal dispersion in less turbid waters.

### 4.1. Magnitude Response

In this section, we show how temporal dispersion is closely linked to multiple scattering and the system’s geometry. Since each photon has been traced independently, the total propagation distance and received power of all the received photons can be easily calculated for each simulation round. This allows calculating the temporal response of the channel. In our model, we have assumed that every photon has some weight and that the total received power is the sum of the photon weights at each scattering event. Every scattering event reduces the weight of the photon by a factor equal to water albedo $w_{o}$. To determine the magnitude response, we form a histogram of received powers to calculate channel bandwidth and thereby understand how the channel spreads light pulses in time. A standard method to analyze temporal response is to measure it in the frequency domain.

The results in Figure 3, Figure 4 and Figure 5 use the calculations for computing the maximum frequency and impulse response presented in Section 3. The magnitude response curves presented contain the received power of photons. As the power of total received photons drops, the magnitude response curves tend to die out quickly. This is associated with the fact that less received photons correspond to fewer values in the received power histogram that estimate the magnitude response.

From Figure 3 and Figure 4 we see that increasing the aperture size and FoV increases the received power gain. Since ocean and coastal waters are less turbid, resulting in fewer scattering events, photons are scattered at relatively larger angles. Therefore, a receiver with a wider aperture and FoV captures a higher number of photons in these waters. The overall power gains are relatively higher than the harbor water because the collected photons are not critically absorbed. It is intuitive based on the nature of the underwater channel and lesser scattering events. It also implies that the multipath attenuation is comparatively lower in the ocean and coastal water than harbor water; thus, the photons have sufficient power to reach the receiver. Hence, the effects of temporal dispersion in the ocean and coastal water can be controlled with wide receivers.

In Figure 5, we see that the change in aperture size and FoV does not affect the channel’s response. It implies that capturing a higher number of photons alone does not help. In harbor water, photons scatter at a shorter distance due to the greater number of scattering events; therefore, received photons are severely scattered and are eventually absorbed. Moreover, smaller scattering angles cause photons to stay close to the optical axis; therefore, wide receivers are not the optimal solution in minimizing multipath attenuation in harbor water. Hence, it is multiple scattering that contributes to the temporal dispersion of varying degrees for all water types.

### 4.2. BER Performance

**Figure 6:**
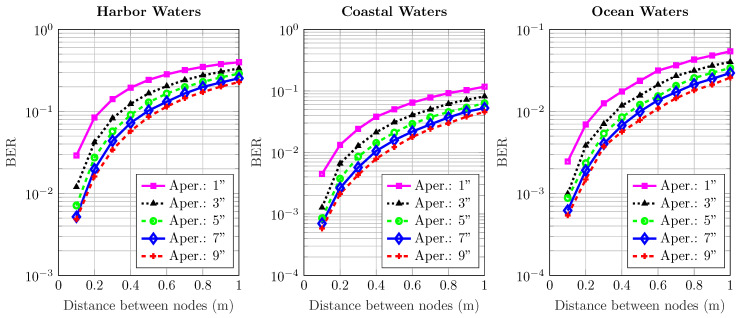
Effect of increasing transmission distance and aperture on the BER performance of harbor, ocean, and coastal waters for 1^o^ receiver FoV.

**Figure 7:**
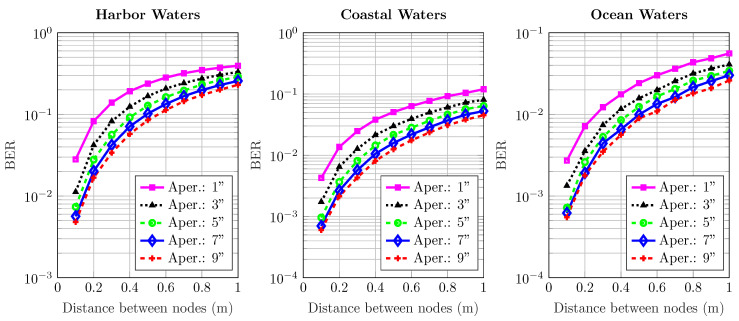
Effect of increasing transmission distance and aperture on the BER performance of harbor, ocean, and coastal waters for 18^o^ receiver FoV.

**Figure 8:**
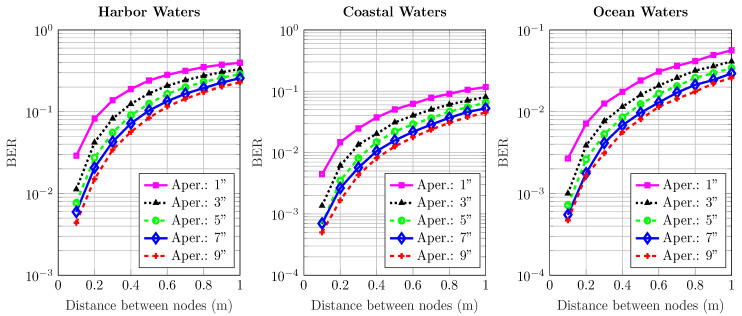
Effect of increasing transmission distance and aperture on the BER performance of harbor, ocean, and coastal waters for 45^o^ receiver FoV.

**Figure 9:**
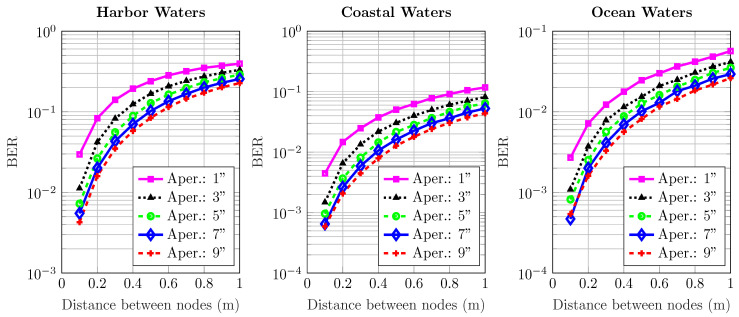
Effect of increasing transmission distance and aperture on the BER performance of harbor, ocean, and coastal waters for 90^o^ receiver FoV.

**Figure 10:**
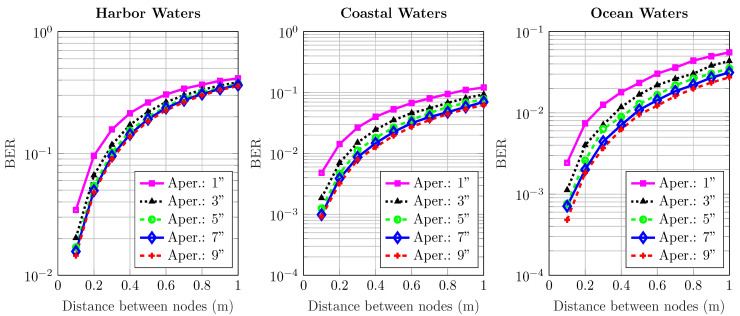
Effect of increasing transmission distance and aperture on the BER performance of harbor, ocean, and coastal waters for 180${}^{\circ }$ receiver FoV.

Figure 6, Figure 7, Figure 8, Figure 9 and Figure 10 depict the BER versus link distance for a range of aperture sizes, FoVs, and different water types. Overall, it can be seen that the BER performance was quite comparable in terms of water types and aperture sizes. The general loss of performance seen in all water types is the result of scattered photons, which transits from low scattering numbers at smaller link distance, to higher scattering numbers at longer link distance. This is an interesting phenomenon, as frequently scattered photons remain close to the optical beam axis, they have a greater probability of intersecting the receiver aperture. However, these photons are heavily absorbed; therefore, they are ultimately rejected upon reception. Furthermore, the results not only show the performance degradation due to temporal dispersion, they also illustrate the loss of performance due to absorption.

The BER performance is improved by the increase of aperture size in harbor, coastal, and ocean water. It is worth noting that the pattern of improvement by increasing apertures is the same for all water types. As the turbidity level increases, so does scattering, which causes severe temporal dispersion. However, as the aperture size increases, BER decreases. In ocean and coastal water, scattering is apt to disperse light over a bigger cross-sectional area due to longer path lengths; thus, scattered photons are less expected to cross the receiver aperture. However, in harbor water, the higher order of scattering events tend to distribute light over a smaller area due to smaller path lengths; therefore, they have a greater chance of crossing the receiver. These results also indicate that the change of FoV is almost negligible. Thus, a wider aperture helps to improve performance, even though minimal, but it shows a connection which is yet to be optimized considering the optimal distance, and optical diffusivity. Greater scattering events not only cause temporal dispersion, they also degrade the photon’s energy as a result of absorption as compared to lesser scattering events which cause less absorption.

### 4.3. Photon Propagation Distance

**Figure 11:**
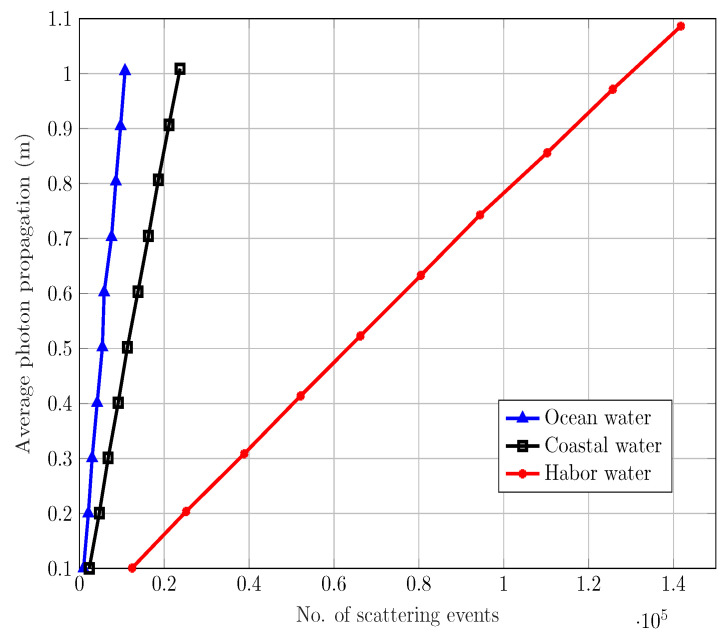
Average distance traveled by photon versus no. of scattering events.

From the magnitude response and BER results for different water types, various apertures, and FoVs, it is clear that as the transmission distance increases, the overall performance and response of the system deteriorates. We have shown that such behavior of different underwater channels is mainly due to different levels of scattering, which upsurges with the increase in water turbidity. From Figure 11, it is clear that with the increase in scattering events the average distance traveled by a photon also increases as the direction vector changes. Consequently, for each scattering event, the average propagation time also increases. Moreover, the figure also shows the actual number of scattering events for the three water types with the highest proportions in harbor waters. These results have been calculated for the same range of distance over which the BER has been calculated. Thus, the overall results suggest that the increase in separation distance between devices contribute to the increase in scattering events and average photon travel distance.

## 5. Discussion

This article proposes a scalable statistical model of wireless optical communication underwater using the Monte Carlo technique. We present the magnitude response of the harbor, coastal, and ocean water by first determining the underwater channel’s impulse response. The baseline model has been adopted from our previous study [10] and is upgraded to be more adaptive and to provide a comprehensive knowledge of the trade-offs in UWOC design. In this regard, the most relevant and state-of-the-art models in the literature are [9,21,27]. Our model estimates the probability of survival of photons from the transmitter to the receiver that can also be referred to as the expected or average value of the underwater optical field under various water conditions and physical limitations. Therefore, the numerical results have been normalized for greater accuracy. We incorporate a dynamic method for calculating bin size of the discrete histograms to (i) reduce the complexity and (ii) increase the efficiency of our model in the measurement of the impulse response. However, the authors in [9] and [27] manually altered the bin sizes to find the right bin width to accommodate the Time-of-Arrival information of the received photons in the histograms. This process is inefficient and tedious at the same time. Therefore, we use a more efficient way of calculating the exact bin width for the discrete histogram of photon received power, known as Scott’s rule. Furthermore, the authors of [9,21] characterized the underwater channel by applying Monte Carlo together with Fournier-Forand (FF), and Sahu and Shanmugam (SS) phase functions, respectively. Whereas, we used Petzold’s measured phase functions along with Monte Carlo to estimate multiple scattering effects. The main findings of this study can be summarized as follows:

- The increase in transmission distance and scattering events increase multipath attenuation.
- Greater scattering and diffusivity of the optical source spreads the received optical power, thus causing temporal dispersion.
- In harbor water, the temporal dispersion is significantly high, and it also affects the data transmission at longer distances.
- A wider aperture and FoV increases received power gains, especially in the ocean and coastal water; however, the effect of FoV alone on overall BER performance is almost negligible in all water types.
- FoV and aperture of an optical wireless communication system underwater are independent of each other; altering one does not necessarily affect the other.

## 6. Conclusions

In this paper, we investigated the temporal spreading of light in underwater wireless optical channels by characterizing the effects of multiple scattering in harbor, coastal, and ocean water. To determine the channel’s IR, we formed a discrete histogram of the total received power normalized by the total received photons. The corresponding magnitude response is calculated by taking the z-transform of the respective channel’s IR. The effects of temporal response can be controlled to some extent by increasing the aperture size in clear and coastal waters; however, for harbor water, the change in receiver parameters has negligible effects on the system’s response. To sum up, greater scattering and diffusivity of the optical source spreads the received optical power, thus causing temporal dispersion.

As part of the future work, our model can accommodate the turbulence effects to acquire a better understanding of channel behavior under all kinds of attenuation sources. In addition, exploring the optimal distances for a required BER can also be a key research problem to solve in UWOC. Our work can benefit in enabling different applications of underwater wearables and can be a possible implementation of full-duplex communication in IoUTs.

## Figures and Tables

**Table 1:** Summary of the advantages and limitations of the three types of evaluation methods.

Study Type	Advantages	Limitations
Simulation-based Modeling	- Flexible - Robust - Capable of imitating the optical propagation loss underwater. - Exploits statistical nature of the optical scattering channel by evaluating a large number of photons (generally $10^{7}$). - Variables, e.g., propagation distance, receiver aperture and FoV can be easily adjusted to real system configurations. - The most general solutions for solving RTE. - Imitates open waters without wall boundaries.	- Time consuming - Results might not be precise. - Reflective waves are usually not modeled.
Experimental Modeling	- Experiments are mostly conducted in a laboratory water tank. - Surface waves can be generated. - Ability to validate the other two methods.	- Measured channel capacity is different from actual underwater channel capacity. - Surface reflective effects are not quantifiable. - Most of the variables of water tank experiment are unalterable, e.g., transmission distance, receiver FoV, aperture, and position. - Initial system set up cost is high. - Ignores the unknown system design aspects, e.g., amount of absorption due to tank wall coatings, and scattering function of the tank walls. - Exact volume scattering function (VSF), initial beam conditions, and water albedo values are usually taken from other published work. - Unable to deduce the effect of transmitter receiver configurations.
Analytical Modeling	- Capable of overcoming the challenges of experimental methods	- Accurate solutions are restrained by a chain of assumptions.

**Table 2:** Petzold’s measured water albedo and attenuation coefficients for the three water types.

Water Type	Attenuation Coefficient *c*	Water Albedo $w_{o}$
Clear	0.15 m${}^{-1}$	0.25
Coastal	0.4 m${}^{-1}$	0.55
Harbor	2.19 m${}^{-1}$	0.83

**Table 3:** Important parameters used for temporal dispersion characterization and MC-based channel simulation.

Coefficients	Symbol	Value
Half-angle beam divergence	$b_{div}$	1.5 mrad
Beam waist	$b_{waist}$	1 mm
Light source wavelength	λ	532 nm
Link distance	*d*	3 m
Water refractive index	$n_{w}$	1.33
Field-of-views	FoV	1${}^{\circ }$, 18${}^{\circ }$, 45${}^{\circ }$, 90${}^{\circ }$, 180${}^{\circ }$
Apertures	$D_{A}$	$1^{\prime \prime }$, $5^{\prime \prime }$, $9^{\prime \prime }$
No. of evaluation points	*n*	512
